# Acute psychosis following corticosteroid administration for COVID-19 and Respiratory Syncytial Virus infection: A case study

**DOI:** 10.1192/j.eurpsy.2023.1668

**Published:** 2023-07-19

**Authors:** E. Miranda Ruiz, E. Marimon Muñoz, J. Ramirez Gonzalez, M. Fariña, R. G. Troyano, M. I. Arroyo Ucar, S. Ferreiro, I. Fernandez Marquez, C. Hidalgo, A. Quispe, L. Delgado

**Affiliations:** 1 Consorci Sanitari de Terrassa, Barcelona

## Abstract

**Introduction:**

Steroids are a necessary treatment for hypoxic respiratory failure; however there are many side effects that should be taken into account. A 44- year-old-woman with asthma and no past psychiatric history was admitted due to COVID-19 pneumonia and Respiratory syncytial virus (RSV) infection, presenting hypoxic respiratory failure. After two days of intravenous methylprednisolone administration, the patient presented acute psychosis and agitation.

It has been previously described that steroid use can cause effects such as mania, anxiety, agitation, delirium and psychosis amongst other. However they are a necessary treatment in respiratory illnesses and are sometimes unavoidable.

**Objectives:**

The aim was to examine the appropriate medical response to steroid induced psychosis in patients with acute hypoxic failure.

**Methods:**

A bibliographical review was done in PubMed database searching recent cases of steroid induced psychosis using the words (“Steroid”, “Psychosis” and “COVID-19”).

**Results:**

According to literature, it has been shown that partial or complete reduction of steroid use and/or use of psychotropic has been successfully used to treat steroid induced psychosis. Following the research it was decided to reduce intravenous methylprednisolone dose from 20mg/ 8h to 20mg/12h and start oral haloperidol 5mg/8h the first 24h and reducing the dose progressively as the patient recovered. After the first 24 hours the patient presented adequate response to steroids as well as partial response to antipsychotic treatment; presenting no further agitation, absence of hallucinations and partial persistence of the persecutory delusion. A couple of days later there was complete remission of the psychotic symptoms and the patient was on the way to recovery from COVID-19 and RSV.

**Conclusions:**

There is evidence that suggests that medications such as steroids used to treat COVID-19 and other respiratory illnesses can lead to psychotic episodes. It is very important to pay attention to possible side effects when treating with steroids and evaluate the patient history as well as suggest having a follow up visit after the hospital discharge.

**Disclosure of Interest:**

None Declared

